# Ovariocytotoxic serum enhances humoral innate immunity and reproductive performance in Kazakh Merino ewes: A controlled field study

**DOI:** 10.14202/vetworld.2026.3850-3863

**Published:** 2026-08-30

**Authors:** Yerganat Korabayev, Abylai Sansyzbay, Kuandyk Shynybayev, Zhuldyzay Kenzhebekova, Khudaibergen Azizov, Almira Ilimbayeva, Ryskeldi Bazarbayev, Askar Terlikbaev, Markhabat Batyrkhanov, Saltanat Nussupova, Assel Zhylgeldiyeva, Ainur Dzhangabulova

**Affiliations:** 1 Department of Pharmacology and Animal Pathology, Kazakh National Agrarian Research University, Almaty, Kazakhstan.; 2 Research Institute of Veterinary Medicine, Pharmacy and Sanitation, Kazakh National Agrarian Research University, Almaty, Kazakhstan.; 3 Kazakh Scientific Research Veterinary Institute, Almaty, Kazakhstan.; 4 Department of Biological Safety, Kazakh National Agrarian Research University, Almaty, Kazakhstan.; 5 Faculty of Veterinary Medicine, Saken Seifullin Kazakh Agrotechnical University, Astana, Kazakhstan.; 6 LLC «Shitemir », Almaty, Kazakhstan.

**Keywords:** artificial insemination, humoral immunity, Kazakh Merino, lamb production, ovariocytotoxic serum, reproductive performance, sheep, twinning

## Abstract

**Background and Aim:**

Improving reproductive efficiency is a major objective in sheep production because increased lamb output directly enhances flock productivity and economic returns. Ovariocytotoxic serum (OCS), an organ-specific hyperimmune preparation produced against ovarian tissue antigens, has been proposed as a biological alternative to conventional hormonal reproductive management. However, its effects on innate humoral immunity and reproductive performance in sheep remain insufficiently characterized. This study evaluated the effects of OCS administration on hematological and humoral immune parameters, estrus and insemination dynamics, and reproductive outcomes in Kazakh Merino ewes under field conditions.

**Materials and Methods:**

A controlled, non-randomized field study was conducted during the breeding season using 200 clinically healthy Kazakh Merino ewes allocated into an OCS-treated group (n = 100) and an untreated control group (n = 100). Treated ewes received two subcutaneous OCS injections before the planned insemination campaign. Hematological indices and serum lysozyme, bactericidal, and complement activities were evaluated in a laboratory subset of 20 ewes per group at five sampling points. Estrus detection, artificial insemination, lambing performance, twinning rate, lamb output, and lamb survival were recorded. Reproductive outcomes were analyzed using Fisher's exact test or chi-square test, and effect estimates were expressed as relative risk (RR), odds ratio, and 95% confidence interval (CI).

**Results:**

OCS administration was associated with descriptive increases in humoral innate immune parameters. Mean lysozyme activity increased from 29.1% to 37.3%, bactericidal activity from 49.4% to 58.8%, and complement activity from 19.8% to 21.6% around estrus. By day 25 of the insemination campaign, 93.0% of OCS-treated ewes had exhibited estrus and been inseminated compared with 46.0% of controls (RR = 2.02; 95% CI: 1.62-2.52; p < 0.0001), reducing the insemination period from 21 to 14 days. Twin lambings were significantly more frequent in the OCS group (32.0% vs. 14.0%; RR = 2.29; 95% CI: 1.30-4.02; p = 0.004), increasing lamb output from 104 to 129 lambs per 100 ewes. Although lambing rate was numerically higher following OCS treatment (97.0% vs. 90.0%), the difference was not statistically significant (p = 0.082).

**Conclusion:**

OCS administration was associated with enhanced humoral innate immune responses, accelerated estrus and insemination dynamics, increased twinning rate, and greater lamb production in Kazakh Merino ewes. These findings support the potential of OCS as a hormone-sparing biological approach for improving reproductive efficiency under field conditions. Nevertheless, randomized placebo-controlled multicenter studies with comprehensive endocrine, immunological, and long-term safety evaluations are required before routine application in sheep breeding programs can be recommended.

## INTRODUCTION

Organ-specific cytotoxic sera have been developed experimentally against various tissues, although only a limited number have been evaluated for practical veterinary applications. Ovariocytotoxic serum (OCS) is a hyperimmune serum raised against ovarian tissue antigens and has been proposed as an organ-specific biological preparation for modulating ovarian function [1–4]. In sheep production, estrus induction and synchronization are conventionally achieved using hormonal protocols based on progestagens, prostaglandins, gonadotropin-releasing hormone (GnRH), equine chorionic gonadotropin/pregnant mare serum gonadotropin (eCG/PMSG), or combinations of these agents. Although these hormonal approaches are generally effective, repeated administration of eCG/PMSG may stimulate the formation of anti-eCG antibodies, thereby reducing the predictability and consistency of reproductive responses in some animals [5–7]. Consequently, there is growing interest in alternative biological strategies to enhance reproductive performance while minimizing reliance on exogenous hormonal treatments.

Among these alternatives, immunomodulatory approaches have received increasing attention. For example, inhibin immunization has been used to increase ovulation rate by modifying the endocrine regulation of follicular development [8, 9]. However, these approaches primarily target endocrine pathways and do not directly address the interaction between innate immune competence and reproductive performance under practical field conditions. Organ-specific cytotoxic sera, including OCS, have been reported to exert dose-dependent stimulatory effects in limited studies involving cattle and in regional veterinary reports. Nevertheless, their influence on humoral non-specific resistance, particularly lysozyme activity, serum bactericidal activity, complement activity, and subsequent reproductive performance in sheep, remains insufficiently characterized [10, 11].

Humoral non-specific resistance constitutes an essential component of innate immunity and provides the first line of defense against invading pathogens. Lysozyme activity, serum bactericidal activity, and complement activity are widely recognized indicators of innate immune status in farm animals because they reflect antimicrobial defense mechanisms and the functional competence of the humoral immune system [12–15]. Among these parameters, complement activity is particularly important because it participates in both antibody-mediated and non-specific antimicrobial responses and may reflect alterations in systemic immune responsiveness during the periovulatory and reproductive periods [16]. Previous investigations have shown that humoral non-specific resistance varies with physiological status, age, pregnancy, lactation, and reproductive stage [17–19]. Studies in ewes have also shown associations between leukocyte count, total serum protein concentration, lysozyme activity, serum bactericidal activity, and natural resistance; however, these immune indicators have rarely been evaluated simultaneously with reproductive performance following OCS administration [20]. Furthermore, although OCS has been associated with changes in lysozyme and serum bactericidal activity in cattle, comparable evidence in sheep remains scarce [21, 22].

Kulataev and Kozhakhmetova [23] reported that stimulatory doses of OCS were associated with improved reproductive performance in ewes. Nevertheless, evidence remains limited because previous studies have generally focused on either reproductive outcomes or selected immune parameters, without comprehensively evaluating concurrent changes under controlled field conditions [24]. In addition, because OCS is produced against ovarian tissue antigens, its administration raises important biological and safety considerations, including the potential for ovarian tissue reactions, autoimmune responses, and long-term effects on reproductive function. These safety aspects have received limited investigation in sheep, highlighting the need for comprehensive studies that simultaneously evaluate both the immunomodulatory and reproductive consequences of OCS administration [25].

Despite previous investigations, several important knowledge gaps remain. Current evidence regarding OCS in sheep is based primarily on regional reports with limited experimental validation, and information describing the simultaneous relationship between humoral non-specific resistance and reproductive performance under practical field conditions is scarce. Moreover, the temporal changes in lysozyme activity, serum bactericidal activity, complement activity, hematological parameters, estrus dynamics, and subsequent lambing performance following OCS administration have not been comprehensively investigated in a single controlled field study. Furthermore, evidence on the potential biological safety of OCS in breeding ewes remains limited, thereby restricting a balanced assessment of its benefits and risks. Addressing these gaps is essential to determine whether OCS represents a biologically plausible and practically applicable hormone-sparing strategy for improving reproductive efficiency in sheep production systems.

Therefore, the present study was designed to evaluate the effects of hyperimmune OCS administration on humoral non-specific resistance and reproductive performance in Kazakh Merino ewes under commercial field conditions. Kazakh Merino ewes were selected because they represent an economically important fine-wool breed in southeastern Kazakhstan, where improving lamb output per 100 ewes is a major production objective. We hypothesized that administration of low-dose OCS before the planned insemination campaign would transiently enhance innate humoral immunity, as reflected by increased lysozyme, serum bactericidal, and complement activities, thereby promoting earlier estrus expression and improved reproductive performance. Specifically, the objectives of this study were to: (1) evaluate changes in hematological parameters and serum lysozyme, bactericidal, and complement activity following OCS administration; (2) assess estrus onset and artificial insemination dynamics in OCS-treated and untreated ewes; and (3) compare lambing rate, barren ewe rate, twinning rate, lamb output per 100 ewes, and lamb survival between the two groups.

## MATERIALS AND METHODS

### Ethical approval

All procedures were conducted in accordance with the Rules for Handling Animals approved by Order of the Minister of Agriculture of the Republic of Kazakhstan dated December 30, 2014 No. 16-02/701 (registered with the Ministry of Justice under No. 10183) [26] and the Law of the Republic of Kazakhstan dated December 30, 2021 No. 97-VII LRK “On Responsible Treatment of Animals” [27] and were guided by the principles of the European Convention for the Protection of Vertebrate Animals used for Experimental and Other Scientific Purposes [28]. 

### Study period and location

The work formed part of a departmental research program conducted from October 2020 to May 2025. The controlled field experiment reported in this manuscript was conducted during the October breeding season at the private farm “Azat,” Rayymbek District, Almaty Region, Kazakhstan. Blood and serum analyses were performed at the Department of Pharmacology and Animal Pathology, Kazakh National Agrarian Research University, Almaty, Kazakhstan.

### Study design

The study evaluated the effects of OCS, produced at the Department of Pharmacology and Animal Pathology, on humoral non-specific resistance and reproductive performance in Kazakh Merino ewes. A non-randomized, analog-selected controlled field design was used under routine farm conditions. According to farm veterinary records and clinical examinations, not clinically apparent infectious or parasitic diseases were detected in the flock during the study period.

### Animals, housing, feeding, and group allocation

A total of 200 clinically healthy Kazakh Merino ewes with a mean live weight of approximately 50 kg were included. The animals were allocated by the analog principle to an OCS-treated group and an untreated control group, each comprising 100 ewes. Group allocation considered age, health status, and body condition.

All ewes were maintained under the same farm-management conditions and received the same ration throughout the experiment. Detailed ration composition and individual body condition scores were unavailable in the archived farm records. The control group did not receive OCS, saline, or another placebo, and blinding was not implemented under the field conditions. The sample size was determined by the number of eligible ewes available during the insemination campaign; no formal *a priori* power calculation was performed.

### Preparation and quality control of OCS

Ovarian tissue collected from slaughtered ewes was used as the antigen for OCS production. The ovaries were trimmed to remove dense connective tissue, washed several times with sterile physiological saline, and homogenized under aseptic conditions. The resulting ovarian tissue homogenate was transferred to sterile containers and stored frozen until use as antigen material [29, 30]. Information on the age and reproductive status of the ovarian tissue donor ewes was unavailable in the archived production records.

Four clinically healthy donor donkeys were used to produce OCS. The donor animals received subcutaneous injections of a 5% ovarian antigen suspension prepared in sterile physiological saline. The antigen was administered in progressively increasing doses at 5–7-day intervals. The cumulative volume of antigen suspension administered during the immunization course was 250–300 mL per donor animal, and no adjuvant was used. The available production records documented the interval between injections and the total administered volume but did not specify the exact number of injections. The sex and exact age of the donor donkeys were also not recorded.

The donor animals were clinically monitored after each antigen administration. Transient depression and reduced feed intake were observed after some injections; however, these signs resolved within 24 h. At 7–8 days after the final antigen injection, a small blood sample was collected from each donor donkey to obtain serum and determine OCS activity using the complement fixation test (CFT). Serum was considered active when the CFT titer was at least 1:100. The OCS batch used in this study had a CFT titer of 1:110 and was therefore considered active according to the departmental production protocol. After activity was confirmed, 500–1000 mL of blood was aseptically collected from the jugular vein of each immunized donor donkey.

The resulting serum underwent routine toxicity testing in white mice and rabbits in accordance with the departmental protocol. After production, the serum was preserved with a 5% phenol solution, allowed to settle for 7 days, dispensed into sterile ampoules, and stored at 4°C until use. Before administration, each ampoule was visually inspected. Preparations showing visible contamination, surface film, mold growth, or an abnormal odor were discarded. Opened ampoules were used on the same day.

Serum preparation was performed under aseptic conditions, and all ampoules were inspected before use. However, data on the final serum protein concentration, endotoxin concentration, sterility culture, purity, and cross-reactivity with non-ovarian tissues were unavailable for the OCS batch used in this study.

### OCS administration protocol

Ewes in the OCS-treated group received two subcutaneous injections of OCS into the middle third of the neck, beginning 2 weeks before the planned insemination campaign. The injections were administered 3 days apart. The first dose was 2.5 mL, and the second dose was 3.0–3.5 mL. Ewes in the control group received neither OCS nor a placebo. The dose regimen was selected in accordance with the departmental production and field-use protocol for stimulatory doses of OCS in livestock.

### Estrus detection and artificial insemination

Ewes in both groups were monitored for estrus throughout the insemination campaign. Estrus was detected through visual observation of behavioral signs and teaser rams. For each ewe, the date of estrus onset, date of artificial insemination, occurrence and date of repeat insemination, and total duration of the insemination campaign were recorded. Artificial insemination was performed according to the routine farm protocol. The archived farm records did not specify whether fresh, chilled, or frozen semen was used.

### Blood sampling

A laboratory subset of 40 ewes, comprising 20 OCS-treated and 20 control animals, was selected for hematological and serum immune analyses. Blood was collected from the jugular vein at five time points: before OCS administration, on day 7 after OCS administration, on the day of estrus, and on days 14 and 21 after artificial insemination.

Samples were collected in the morning before feeding from clinically healthy ewes. Before venipuncture, the sampling site was clipped and disinfected with 96% ethanol. Sterile, dry needles and collection tubes were used. Blood intended for hematological analysis was anticoagulated with heparin at a final concentration of 5 IU/mL. Blood and serum samples were stored under refrigerated conditions, and laboratory analyses were performed on the following day.

### Laboratory analyses

Humoral non-specific resistance was evaluated by measuring serum lysozyme, bactericidal, and complement activities. Hematological and biochemical measurements included erythrocyte count, leukocyte count, hemoglobin concentration, and total serum protein concentration. Total serum protein was determined refractometrically. Erythrocyte count, leukocyte count, and hemoglobin concentration were determined using the routine hematological procedures of the Department of Pharmacology and Animal Pathology.

### Serum lysozyme activity

Serum lysozyme activity was determined using the photocolorimetric method of Dorofeychuk, with *Micrococcus lysodeikticus* as the reference organism. A 24-h culture of *M. lysodeikticus* was suspended in phosphate buffer at pH 7.4, filtered to remove aggregates, and standardized photocolorimetrically using a green filter and a 10-mm cuvette.

For the assay, 1.47 mL of the standardized microbial suspension was mixed with 0.03 mL of fresh serum, yielding a serum dilution of 1:50. The mixture was incubated at 37°C for 1 h and subsequently measured photocolorimetrically under identical conditions. Lysozyme activity was expressed as the percentage change in light transmittance of the test suspension relative to that of the initial microbial suspension.

### Serum complement activity

Serum complement activity was determined photocolorimetrically based on the hemolysis of sensitized sheep erythrocytes. Serum was separated after clotting by centrifugation at 1,500 rpm (approximately 377 × *g*) for 12 min. A 3% suspension of washed ram erythrocytes was prepared in isotonic saline and standardized photocolorimetrically.

The hemolytic system consisted of equal volumes of the 3% erythrocyte suspension and diluted hemolytic serum. The mixture was incubated at 37°C for 30 min to sensitize the erythrocytes. For each test sample, 0.1 mL of serum was mixed with 5.9 mL of physiological saline and 4 mL of the hemolytic system. A control tube containing distilled water instead of physiological saline represented complete hemolysis. After incubation at 37°C for 30 min, the tubes were centrifuged at 3,000 rpm (approximately 1,509 × *g*) for 10 min, and the optical density (OD) of the supernatant was measured photocolorimetrically. Complement activity was calculated as follows:

K (%) = (E₀/Eₖ) × 100

where K is the percentage of hemolysis, E₀ is the OD of the test sample, and Eₖ is the OD of the complete hemolysis control.

### Serum bactericidal activity

Serum bactericidal activity was determined using the photonephelometric method modified by Smirnova and Kuzmina [31], which is based on the inhibitory effect of serum on bacterial growth in a nutrient medium. *Escherichia coli* strain No. 157 was used as the reference organism.

The test mixture contained 4.5 mL of meat-peptone broth, 1.0 mL of test serum, and one drop of a 24-h *E. coli* culture containing approximately 1 × 10⁹ microbial cells/mL. The control mixture contained 4.5 mL of meat-peptone broth, 1.0 mL of physiological saline, and one drop of the same *E. coli* culture. Baseline measurements were obtained photocolorimetrically using a green filter and a 5-mm cuvette. The mixtures were then incubated at 37.5°C for 3 h and measured again under the same conditions. The change in OD after incubation represented microbial growth in the test and control mixtures. Serum bactericidal activity was calculated as follows:

Bactericidal activity (%) = [1 − (ΔODₜₑₛₜ/ΔODcₒₙₜᵣₒₗ)] × 100

where ΔODₜₑₛₜ is the change in OD of the serum-containing test mixture after 3 h of incubation and ΔODcₒₙₜᵣₒₗ is the corresponding change in the control mixture. Higher bactericidal activity values indicated greater serum-mediated inhibition of bacterial growth.

### Definitions of reproductive outcomes

Reproductive performance was evaluated using estrus, insemination, lambing, and lamb survival records. Estrus response was defined as the number of ewes detected in estrus during the insemination campaign. Lambing rate was calculated as the number of ewes that lambed, divided by the number of ewes assigned to the group, and expressed as a percentage. Barren ewe rate was calculated as the number of ewes that did not lamb divided by the number of ewes assigned to the group.

Twinning rate was defined as the number of ewes with twin lambings divided by the total number of ewes assigned to the group. Lamb output per 100 ewes was calculated as the number of lambs born divided by the number of ewes assigned to the group, multiplied by 100. Lamb mortality was calculated as the number of lambs that died after birth divided by the total number of lambs born. Lamb survival was calculated as the number of surviving lambs divided by the total number of lambs born. The numbers of abortions and stillbirths were obtained from the farm lambing records.

Because pregnancy diagnosis by ultrasonography or rectal palpation was not performed consistently across the study population, breeding success was assessed using lambing records. Therefore, the term “lambing rate,” rather than “pregnancy rate” or “fertility rate,” was used to describe reproductive success.

### Clinical observations

After OCS administration, ewes were monitored for visible local and systemic adverse reactions, including injection-site swelling, depression, reduced feed intake, abortion, stillbirth, lambing complications, and delayed placental separation. Farm personnel and veterinary staff conducted clinical observations during the insemination, gestation, and lambing periods. Findings were summarized from the available farm records.

### Statistical analysis

Statistical analyses were performed using R software, version 4.6.1 (R Foundation for Statistical Computing, Vienna, Austria). Estrus-related and reproductive outcomes were analyzed using complete individual outcome records for all 200 ewes included in the field experiment, comprising 100 OCS-treated ewes and 100 untreated controls.

Categorical outcomes, including estrus response, lambing rate, barren ewe rate, twinning rate, lamb mortality, and lamb survival, were compared between groups using Fisher’s exact test or the chi-square test, as appropriate according to expected cell frequencies. The distribution of estrus and insemination timing categories was compared between groups using the chi-square test. Lambing outcomes for individual ewes were categorized as 0, 1, or 2 lambs born, and the resulting distributions were also compared using the chi-square test.

Effect estimates were reported as absolute difference, relative risk (RR), odds ratio (OR), and 95% confidence interval (CI), where applicable. Lamb output was expressed as both the number of lambs born per 100 ewes assigned to each group and the mean number of lambs born per ewe. Absolute and relative between-group differences were calculated for the principal reproductive outcomes. All p-values were two-sided, and p < 0.05 was considered statistically significant.

Hematological and serum immune parameters were evaluated in the laboratory subset of 20 ewes per group. Because individual-level repeated measurements and a verified definition of the reported dispersion measure were unavailable, laboratory data were summarized descriptively at each sampling point using group means, absolute changes, percentage changes from baseline, and between-group differences. No inferential statistical comparisons were performed for these laboratory outcomes.

## RESULTS

### Hematological and serum biochemical dynamics

Hematological and serum biochemical parameters were summarized descriptively for the laboratory subset of 40 ewes (20 per group). Baseline mean values for total serum protein, erythrocyte count, leukocyte count, and hemoglobin concentration were generally comparable between the OCS-treated and control groups. Following OCS administration, all four parameters increased more markedly in the treated group, with the highest mean values observed on the day of estrus (Table 1). Because individual-level repeated measurements and a verified definition of the reported error measure were unavailable, we did not perform inferential statistical comparisons for these laboratory outcomes.

Mean total serum protein in the OCS-treated group increased from 70.1 g/L at baseline to 74.9 g/L on day 7 after OCS administration and reached 88.8 g/L on the day of estrus, representing a 26.7% increase from baseline. The value subsequently declined to 74.7 g/L on day 14 and 70.4 g/L on day 21 after artificial insemination. In the control group, total serum protein increased from 70.3 g/L at baseline to 76.1 g/L on the day of estrus, corresponding to an 8.3% increase, before declining to 71.6 g/L by day 21 after artificial insemination.

Mean erythrocyte count in the OCS-treated group increased from 7.1 × 10¹²/L at baseline to 8.2 × 10¹²/L on day 7 and 9.3 × 10¹²/L on the day of estrus, corresponding to a 31.0% increase from baseline. The count subsequently declined to 8.3 × 10¹²/L on day 14 and 7.9 × 10¹²/L on day 21 after artificial insemination. In the control group, erythrocyte count increased from 7.2 × 10¹²/L at baseline to 7.7 × 10¹²/L on the day of estrus, representing a 6.9% increase, and remained at 7.6 × 10¹²/L on days 14 and 21 after artificial insemination.

Mean leukocyte count in the OCS-treated group increased from 7.1 × 10⁹/L at baseline to 8.5 × 10⁹/L on day 7 and 10.9 × 10⁹/L on the day of estrus, representing a 53.5% increase from baseline. Although the value subsequently declined, it remained above baseline at 9.7 × 10⁹/L and 9.6 × 10⁹/L on days 14 and 21 after artificial insemination, respectively. In the control group, leukocyte count increased from 6.9 × 10⁹/L at baseline to 7.5 × 10⁹/L on the day of estrus, corresponding to an 8.7% increase, and remained at approximately 7.4 × 10⁹/L thereafter.

Mean hemoglobin concentration in the OCS-treated group increased from 75.6 g/L at baseline to 82.05 g/L on day 7 and 83.96 g/L on the day of estrus, corresponding to an 11.1% increase from baseline. It subsequently declined to 79.16 g/L on day 14 and 78.2 g/L on day 21 after artificial insemination. In the control group, hemoglobin concentration changed only slightly, increasing from 73.51 g/L at baseline to 74.26 g/L on the day of estrus, corresponding to an increase of approximately 1.0%.

Overall, these descriptive laboratory profiles indicated a transient increase in hematological and serum biochemical parameters following OCS administration, with the most pronounced changes occurring around estrus. Total serum protein returned close to baseline by day 21 after artificial insemination, whereas erythrocyte count, leukocyte count, and hemoglobin concentration remained modestly above their respective baseline values in the OCS-treated group.

**Table 1:** Hematological and serum biochemical parameters in OCS-treated and control Kazakh Merino ewes at different sampling points.

**Parameter**	**Unit**	**Group**	**Baseline**	**Day 7 after OCS administration**	**Estrus** ** day**	**Day 14 after ** **AI**	**Day 21 after ** **AI**
Total protein	g/L	OCS-treated	70.1 ± 0.14	74.9 ± 0.11	88.8 ± 0.09	74.7 ± 0.06	70.4 ± 0.09
		Control	70.3 ± 0.14	71.6 ± 0.13	76.1 ± 0.09	72.9 ± 0.08	71.6 ± 0.08
Erythrocytes	×10¹²/L	OCS-treated	7.1 ± 0.24	8.2 ± 0.32	9.3 ± 0.32	8.3 ± 0.33	7.9 ± 0.31
		Control	7.2 ± 0.22	7.4 ± 0.24	7.7 ± 0.21	7.6 ± 0.22	7.6 ± 0.32
Leukocytes	×10⁹/L	OCS-treated	7.1 ± 0.30	8.5 ± 0.37	10.9 ± 0.32	9.7 ± 0.53	9.6 ± 0.32
		Control	6.9 ± 0.20	7.2 ± 0.32	7.5 ± 0.30	7.4 ± 0.32	7.4 ± 0.31
Hemoglobin	g/L	OCS-treated	75.6 ± 2.16	82.05 ± 1.96	83.96 ± 2.03	79.16 ± 2.13	78.2 ± 1.86
		Control	73.51 ± 1.16	74.10 ± 1.94	74.26 ± 2.12	74.21 ± 1.89	74.20 ± 1.94

Values are presented as mean ± reported error from the archived laboratory dataset. Laboratory analyses were performed in a subset of 20 ewes per group. OCS = Ovariocytotoxic serum; AI = Artificial insemination.

### Humoral non-specific resistance

Serum lysozyme, bactericidal, and complement activities showed more pronounced descriptive increases in the OCS-treated group than in the control group (Table 2). Baseline mean values were generally similar between groups, whereas the largest between-group differences were observed on the day of estrus. These laboratory findings were interpreted descriptively because the archived dataset did not provide individual-level repeated measurements or clarify whether the reported error represented the standard deviation or standard error.

Mean serum lysozyme activity in the OCS-treated group increased from 29.1% at baseline to 33.27% on day 7 after OCS administration and reached 37.32% on the day of estrus, corresponding to a 28.2% increase from baseline. Lysozyme activity remained elevated at 36.15% on day 14 and 34.05% on day 21 after artificial insemination. In the control group, lysozyme activity increased from 28.9% at baseline to 30.13% on the day of estrus, representing a 4.3% increase, and subsequently declined to 29.12% by day 21 after artificial insemination.

Mean serum bactericidal activity in the OCS-treated group increased from 49.4% at baseline to 52.2% on day 7 and 58.8% on the day of estrus, corresponding to a 19.0% increase from baseline. The value subsequently declined but remained above baseline at 55.5% on day 14 and 52.7% on day 21 after artificial insemination. In the control group, bactericidal activity increased from 49.8% at baseline to 51.2% on the day of estrus and remained within a narrow range throughout the observation period.

Complement activity showed a smaller descriptive response. In the OCS-treated group, mean complement activity increased from 19.8% at baseline to 20.8% on day 7 and 21.6% on the day of estrus, representing a 9.1% increase from baseline. It subsequently declined slightly to 21.0% on day 14 and 20.6% on day 21 after artificial insemination. In the control group, complement activity remained nearly unchanged, increasing from 19.9% at baseline to 20.0% on the day of estrus and declining to 19.8% on days 14 and 21 after artificial insemination.

**Figure 1:**
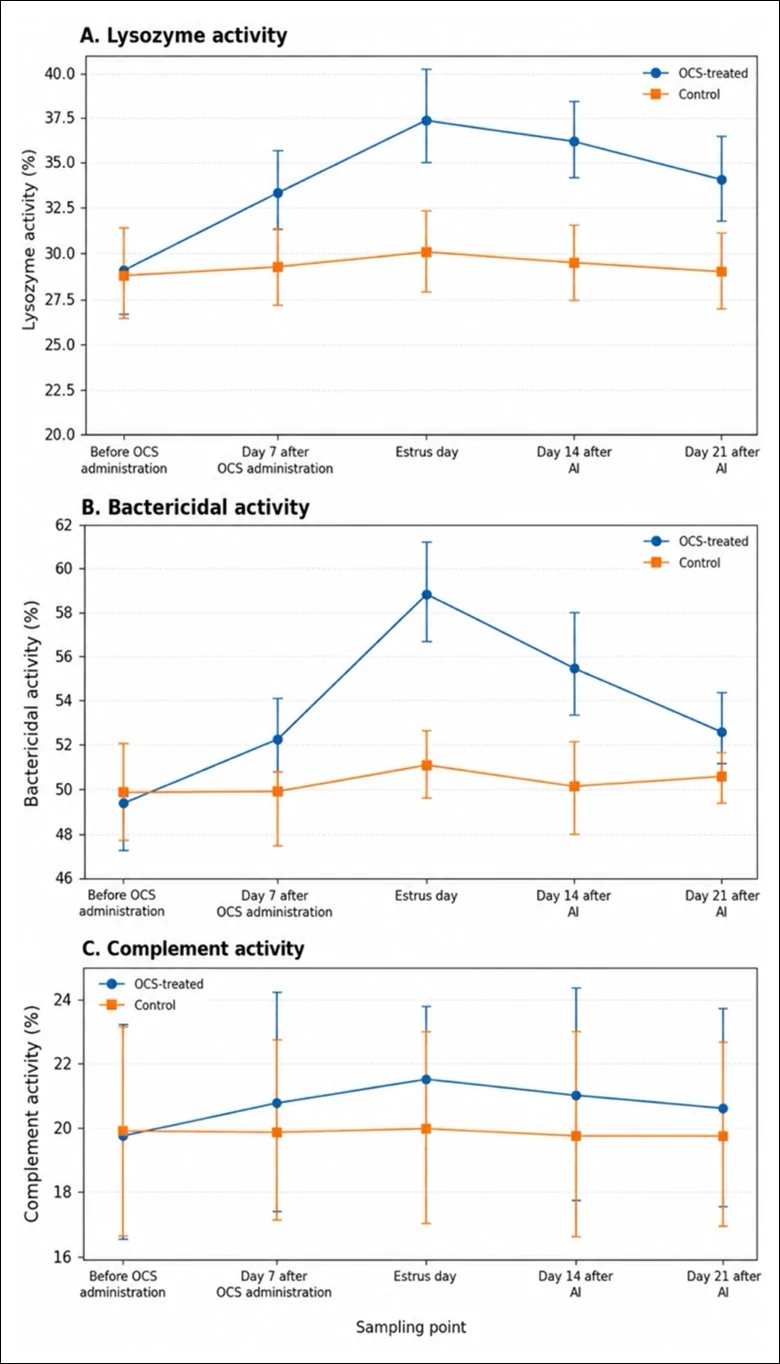
Dynamics of serum humoral non-specific resistance parameters in OCS-treated and control Kazakh Merino ewes. (A) Lysozyme activity; (B) bactericidal activity; and (C) complement activity. Values are presented as mean ± reported error from the archived laboratory dataset. Laboratory analyses were performed in a subset of 20 ewes per group. The archived dataset did not specify whether the reported error was the standard deviation or the standard error; therefore, these data were interpreted descriptively, and no inferential statistical measures were included. OCS = Ovariocytotoxic serum.

Figure 1 presents the temporal patterns of serum lysozyme, bactericidal, and complement activities. Collectively, these findings indicated that OCS administration was followed by a transient descriptive increase in selected humoral non-specific resistance parameters, particularly lysozyme and bactericidal activities, around the time of estrus (Table 2; Figure 1).

**Table 2:** Serum humoral non-specific resistance parameters in OCS-treated and control Kazakh Merino ewes.

**Parameter**	**Unit**	**Group**	**Baseline**	**Day 7 after OCS administration**	**Estrus** ** day**	**Day 14 after ** **AI**	**Day 21 after ** **AI**
Lysozyme activity	%	OCS-treated	29.1 ± 2.23	33.27 ± 2.26	37.32 ± 2.34	36.15 ± 1.97	34.05 ± 2.29
		Control	28.9 ± 2.30	29.33 ± 2.00	30.13 ± 2.11	29.57 ± 2.06	29.12 ± 2.03
Bactericidal activity	%	OCS-treated	49.4 ± 2.27	52.2 ± 1.58	58.8 ± 2.25	55.5 ± 2.00	52.7 ± 1.50
		Control	49.8 ± 2.03	49.9 ± 2.46	51.2 ± 1.50	50.2 ± 1.96	50.6 ± 1.10
Complement activity	%	OCS-treated	19.8 ± 3.30	20.8 ± 3.40	21.6 ± 2.20	21.0 ± 3.33	20.6 ± 3.10
		Control	19.9 ± 3.30	19.9 ± 2.80	20.0 ± 3.00	19.8 ± 3.22	19.8 ± 2.90

Values are presented as mean ± reported error from the archived laboratory dataset. Laboratory analyses were performed in a subset of 20 ewes per group. OCS = Ovariocytotoxic serum; AI = Artificial insemination.

### Estrus and artificial insemination dynamics

All ewes in both groups were eventually detected in estrus and inseminated during the insemination campaign; however, the timing of estrus detection and artificial insemination differed markedly between groups (Table 3). In the OCS-treated group, 33 ewes were detected in estrus and inseminated between days 15 and 20, 60 between days 21 and 25, and seven between days 26 and 30. No OCS-treated ewe required 31 days or longer to reach estrus and undergo insemination. In the control group, 12 ewes were detected and inseminated between days 15 and 20, 34 between days 21 and 25, 40 between days 26 and 30, and 14 on or after day 31. The overall distribution of estrus and insemination timing differed significantly between groups (χ² = 54.16, df = 3, p < 0.0001).

By day 25, 93 of 100 OCS-treated ewes had been detected in estrus and inseminated, compared with 46 of 100 control ewes. Thus, OCS-treated ewes were approximately twice as likely to have been detected and inseminated by day 25 as control ewes (RR = 2.02; 95% CI: 1.62–2.52). The corresponding OR was 15.60 (95% CI: 6.58–36.97), and the difference was statistically significant (Fisher’s exact p < 0.0001).

By day 30, all 100 OCS-treated ewes had been detected in estrus and inseminated, compared with 86 of 100 control ewes (100.0% vs. 86.0%; Fisher’s exact p < 0.001). The insemination campaign lasted 14 days in the OCS-treated group and 21 days in the control group, indicating a 7-day reduction in campaign duration following OCS administration (Table 3).

**Table 3:** Estrus detection and artificial insemination timing in OCS-treated and control Kazakh Merino ewes.

**Group**	**Ewes ** **(n)**	**Days** **15–20**	**Days** **21–25**	**Days** **26–30**	**≥31** **days**	**Detected/inseminated ** **by day 25**	**Detected/inseminated by day 30**	**Insemination campaign duration**
OCS-treated	100	33	60	7	0	93	100	14 days
Control	100	12	34	40	14	46	86	21 days

OCS = Ovariocytotoxic serum. The distribution of estrus/insemination timing categories differed between groups (χ² = 54.16, df = 3, p < 0.0001). For detection/insemination within 25 days, RR = 2.02, 95% CI: 1.62–2.52; Fisher’s exact p < 0.0001. For detection/insemination within 30 days, Fisher’s exact p < 0.001.

### Lambing outcomes and lamb survival

Overall, 187 of the 200 ewes lambed, resulting in an overall lambing rate of 93.5% (Table 4). Thirteen ewes remained barren. A total of 233 lambs were born from 141 single and 46 twin lambings, and seven lambs died after birth. No abortions or stillbirths were documented in the available farm lambing records.

In the OCS-treated group, 97 of 100 ewes lambed, compared with 90 of 100 ewes in the control group, representing an absolute difference of 7 percentage points. Although the odds of lambing were higher in the OCS-treated group, the difference did not reach statistical significance (OR = 3.59; 95% CI: 0.96–13.47; Fisher’s exact p = 0.082). Correspondingly, three OCS-treated ewes and 10 control ewes remained barren, resulting in barren ewe rates of 3.0% and 10.0%, respectively (OR = 0.28; 95% CI: 0.07–1.04; Fisher’s exact p = 0.082).

Twin lambings occurred more frequently in the OCS-treated group. Thirty-two of 100 OCS-treated ewes produced twins, compared with 14 of 100 control ewes. This represented an absolute increase of 18 percentage points and a 2.29-fold higher probability of twin lambing in the OCS-treated group (RR = 2.29; 95% CI: 1.30–4.02). The corresponding OR was 2.89 (95% CI: 1.43–5.84), and the difference was statistically significant (Fisher’s exact p = 0.004).

Among ewes that lambed, 32 of 97 OCS-treated ewes produced twins, compared with 14 of 90 control ewes, corresponding to twinning rates of 33.0% and 15.6%, respectively. OCS-treated ewes that lambed were therefore more than twice as likely to produce twins as control ewes (RR = 2.12; 95% CI: 1.21–3.71; OR = 2.67; 95% CI: 1.31–5.44; Fisher’s exact p = 0.0066).

A total of 129 lambs were born in the OCS-treated group, compared with 104 lambs in the control group. Lamb output was 129 and 104 lambs per 100 ewes in the respective groups, representing an increase of 25 lambs per 100 ewes, or 24.0%, following OCS administration. The mean number of lambs born per ewe assigned to the group was 1.29 in the OCS-treated group and 1.04 in the control group (Mann–Whitney U test, p = 0.00068).

The distribution of lambing outcomes also differed between groups. In the OCS-treated group, three ewes produced no lambs, 65 produced one lamb, and 32 produced two lambs. In the control group, 10 ewes produced no lambs, 76 produced one lamb, and 14 produced two lambs. The distribution of zero, one, or two lambs per ewe differed significantly between groups (χ² = 11.67, df = 2, p = 0.0029).

Lamb mortality was numerically lower in the OCS-treated group. Two of 129 lambs born in the OCS-treated group died after birth, compared with five of 104 lambs in the control group, corresponding to mortality rates of 1.6% and 4.8%, respectively. However, this difference was not statistically significant (RR = 0.32; 95% CI: 0.06–1.63; OR = 0.31; 95% CI: 0.06–1.64; Fisher’s exact p = 0.247). Lamb survival rates were therefore 98.4% in the OCS-treated group and 95.2% in the control group (Table 4).

**Table 4:** Lambing outcomes and lamb survival in OCS-treated and control Kazakh Merino ewes.

**Outcome**	**OCS-treated**	**Control**	**Effect estimate/statistical analysis**
Ewes assigned to group (n)	100	100	—
Ewes lambed, n (%)	97 (97.0)	90 (90.0)	OR = 3.59 (95% CI: 0.96–13.47); Fisher's exact p = 0.082
Barren ewes, n (%)	3 (3.0)	10 (10.0)	OR = 0.28 (95% CI: 0.07–1.04); Fisher's exact p = 0.082
Single lambings (n)	65	76	—
Twin lambings, n (%)	32 (32.0)	14 (14.0)	RR = 2.29 (95% CI: 1.30–4.02); OR = 2.89 (95% CI: 1.43–5.84); Fisher's exact p = 0.004
Twinning rate among lambed ewes	32/97 (33.0%)	14/90 (15.6%)	RR = 2.12 (95% CI: 1.21–3.71); OR = 2.67 (95% CI: 1.31–5.44); Fisher's exact p = 0.0066
Total lambs born (n)	129	104	+25 lambs per 100 ewes (+24.0%)
Lambs born per ewe assigned (mean)	1.29	1.04	Mann–Whitney U test, p = 0.00068
Lambing outcome (0/1/2 lambs per ewe)	3/65/32	10/76/14	χ² = 11.67, df = 2, p = 0.0029
Surviving lambs, n (%)	127/129 (98.4)	99/104 (95.2)	Fisher's exact p = 0.247
Dead lambs after birth, n (%)	2/129 (1.6)	5/104 (4.8)	RR = 0.32 (95% CI: 0.06–1.63); Fisher's exact p = 0.247

OCS = Ovariocytotoxic serum; RR = Relative risk; OR =Odds ratio; CI =Confidence interval. Twinning rate was calculated as the number of ewes with twin lambings divided by the number of ewes assigned to the group, unless otherwise stated. Lambs per 100 ewes refers to the total number of lambs born per 100 ewes assigned to the group, not the number of lambs weaned.

## DISCUSSION

### OCS improves reproductive performance under field conditions

The present controlled field study demonstrated that OCS administration was associated with earlier estrus onset, accelerated insemination dynamics, descriptive enhancement of humoral non-specific resistance, and improved reproductive performance in Kazakh Merino ewes. The principal reproductive benefits were a significantly higher twinning rate, greater lamb output per 100 ewes, and a shorter insemination campaign. Although the lambing rate was numerically higher in the OCS-treated group than in the control group, this difference did not reach statistical significance according to Fisher's exact test. Therefore, the reproductive benefits observed in the present study should be interpreted primarily as increased prolificacy and improved breeding efficiency rather than a statistically confirmed increase in overall lambing rate.

These findings support the growing recognition that reproductive function is closely linked with immune regulation. Sahu *et al.* [32] emphasized that key reproductive events, including ovulation, implantation, and gestation, are coordinated through complex interactions among immune cells, cytokines, endocrine signals, and reproductive tissues. Disturbances in this immune-endocrine network may contribute to inflammatory reproductive disorders, embryonic loss, and immune-mediated infertility. Consequently, immunomodulatory interventions can influence ovarian activity and reproductive performance, although their efficacy depends on treatment dose, timing, physiological status, and species-specific responses.

### Effects of OCS on humoral non-specific resistance

The laboratory findings suggested a descriptive enhancement of humoral non-specific resistance following OCS administration. Because individual-level repeated laboratory measurements were unavailable, these data were interpreted descriptively rather than inferentially. The OCS-treated group consistently exhibited higher mean lysozyme and serum bactericidal activities at the estrus sampling point than the control group, whereas complement activity showed only modest changes. Korabayev *et al.* [25] reported similar descriptive increases in hematological and serum immune parameters following subcutaneous OCS administration in ewes. Nevertheless, the present study does not permit robust statistical conclusions regarding laboratory responses because individual repeated measurements and a verified measure of dispersion were unavailable.

### Reproductive responses following OCS administration

The reproductive findings provided stronger statistical evidence than the laboratory observations. The most notable response was a significant increase in the twinning rate, from 14.0% in the control group to 32.0% following OCS administration, representing a 2.29-fold increase in RR. Likewise, lamb output increased from 104 to 129 lambs per 100 ewes, corresponding to an additional 25 lambs per 100 ewes.

Previous regional studies involving sheep have reported comparable improvements in reproductive performance following OCS administration, including improvements in estrus-related parameters and reproductive hormone profiles [23, 33]. Collectively, these findings indicate that the principal practical benefit of OCS lies in improving prolificacy and flock productivity rather than substantially increasing the proportion of ewes that successfully lamb.

### Possible mechanisms underlying the biological response

The apparent stimulatory effect of a preparation described as "cytotoxic" requires careful biological interpretation. One plausible explanation is that administration of low-dose ovarian antigens induces controlled immunomodulation rather than destructive ovarian injury. Such stimulation may involve localized inflammatory signaling, cytokine-mediated communication, antigen-dependent immune activation, or indirect regulation of follicular development. This interpretation is consistent with the hormesis-like, dose-dependent biological responses proposed for other biologically active preparations [34].

Nevertheless, the present study did not evaluate cytokine production, macrophage activation, ovarian histopathology, ovarian reserve, or anti-ovarian antibody responses. Consequently, the underlying biological mechanisms remain speculative and require confirmation through mechanistic investigations.

### Comparison with other reproductive immunomodulatory strategies

The present findings should also be interpreted alongside other immunological approaches used to enhance reproductive performance in small ruminants. Ullah *et al.* [35] demonstrated that active inhibin immunization increases ovulation rate by suppressing inhibin-mediated negative feedback on follicle-stimulating hormone, thereby promoting follicular development. A previous study targeting ovarian or steroid-related antigens have similarly reported improvements in ovulation rate, although some immunization strategies were associated with reduced mating behavior or conception rates [36].

Unlike inhibin immunization, OCS is not directed against a single endocrine hormone; it is an organ-specific hyperimmune preparation raised against ovarian tissue antigens. Consequently, OCS should not be regarded as biologically equivalent to hormonal synchronization protocols or inhibin immunization. Instead, it may represent a distinct hormone-sparing biological strategy whose efficacy and mechanism require further validation.

### Relationship to conventional estrus synchronization protocols

Conventional estrus synchronization in sheep production relies primarily on progestagens, prostaglandins, GnRH, eCG/PMSG, or combinations of these hormones. Although these protocols are generally effective, repeated administration of eCG/PMSG may induce anti-eCG antibody production and reduce treatment predictability in some animals [37].

The present study does not demonstrate the superiority of OCS over established hormonal synchronization protocols because it did not include a direct comparative treatment group. Nevertheless, the observed shortening of the insemination campaign together with increased lamb output suggests that OCS warrants further investigation as a potential hormone-sparing reproductive management strategy.

### Safety considerations

Safety remains an important consideration for any ovarian-derived hyperimmune preparation. No abortions or stillbirths were recorded in either treatment group, and lamb mortality was numerically lower following OCS administration. However, the present study was not specifically designed to evaluate product safety.

The study did not systematically investigate potential local injection-site reactions, systemic adverse events, long-term ovarian function, ovarian reserve, repeated administration, tissue residues, food safety, or autoimmune responses against ovarian tissue. Previous studies have demonstrated that repeated immunization may produce systemic or reproductive effects under certain circumstances [37, 38]. Therefore, the present findings should not be interpreted as evidence of long-term safety. Future investigations should incorporate structured adverse-event monitoring, endocrine profiling, ovarian ultrasonography, ovarian reserve assessment, and long-term follow-up over multiple breeding seasons.

### Practical implications

The increase in lamb output from 104 to 129 lambs per 100 ewes may have important practical implications for commercial sheep production, particularly where flock productivity largely determines economic return. OCS has already been applied under field conditions on several farms within the Almaty region, including Ili and Rayymbek districts [39–41].

However, this study did not include a formal economic evaluation. Consequently, conclusions regarding cost-effectiveness remain preliminary. Future studies should incorporate comprehensive economic analyses that include serum production, donor animal maintenance, antigen preparation, quality control, labor, administrative costs, animal monitoring, and improvements in reproductive performance.

### Strengths and limitations of the study

The principal strength of the present study is the combined evaluation of humoral non-specific resistance and reproductive performance following OCS administration under practical commercial field conditions. Previous investigations have largely focused on either cattle or regional veterinary applications, whereas evidence simultaneously evaluating immune and reproductive responses in sheep remains limited [39–41]. The present study therefore provides novel evidence linking changes in lysozyme activity, bactericidal activity, complement activity, estrus dynamics, twinning rate, and lamb output.

Nevertheless, several limitations should be acknowledged. The study employed a non-randomized, analog-selected controlled field design on a single commercial farm. The control animals did not receive placebo treatment, and blinding was not implemented. Individual-level repeated laboratory measurements were unavailable, and the reported measure of dispersion could not be confirmed as either standard deviation or standard error. Pregnancy diagnosis was based on lambing records rather than ultrasonography or rectal palpation. Furthermore, information regarding birth weight, lamb sex, weaning performance, parity, body condition score, reproductive history, final serum protein concentration, endotoxin concentration, sterility testing, purity assessment, and cross-reactivity with non-ovarian tissues was unavailable.

### Future research directions

Future investigations should validate these findings through randomized, placebo-controlled, blinded multicenter field trials involving multiple sheep breeds and breeding seasons. Further studies should also include individual-level laboratory measurements, endocrine profiling, ovarian ultrasonography, anti-ovarian antibody determination, cytokine analyses, comprehensive lamb performance records, structured safety assessments, and dose-optimization studies. Such investigations will be essential to determine whether OCS can be incorporated safely and reliably into practical sheep reproduction programs.

## CONCLUSION

This controlled field study demonstrated that OCS administration was associated with improvements in both humoral innate immunity and reproductive performance in Kazakh Merino ewes. OCS-treated animals exhibited descriptive increases in serum lysozyme, bactericidal, and complement activities during the peri-estrus period, together with more synchronized estrus expression and earlier completion of the insemination campaign. Reproductive benefits were reflected by a significantly higher twinning rate, increased lamb output from 104 to 129 lambs per 100 ewes, and a numerically higher lambing rate compared with untreated controls, indicating that the principal advantage of OCS was enhanced prolificacy rather than a statistically significant increase in the proportion of ewes that lambed.

From a practical perspective, these findings suggest that OCS may represent a promising hormone-sparing biological approach for improving reproductive efficiency in sheep production systems. Earlier, more synchronized estrus can facilitate breeding management, shorten insemination campaigns, and improve labor efficiency, whereas increased twinning and lamb output may enhance flock productivity and economic returns. If confirmed under diverse production conditions, OCS could complement existing reproductive management strategies, particularly in systems seeking alternatives to repeated hormonal synchronization protocols.

Overall, these findings support continued investigation of OCS as an innovative immunomodulatory strategy with the potential to improve reproductive efficiency and flock productivity, while emphasizing that broader validation is required before routine field application can be recommended.

## DATA AVAILABILITY

The data supporting the findings of this study are available from the corresponding authors upon reasonable request.

## GENERATIVE AI DECLARATION

The authors declare that no generative artificial intelligence (AI) tools were used in the preparation, writing, editing, or revision of this manuscript.

## AUTHORS’ CONTRIBUTIONS

YK: Conceptualization, methodology, investigation, supervision, and writing – original draft preparation. AS: Investigation, data curation, and writing – review and editing. KS: Laboratory analysis, validation, and methodology. ZK: Investigation, laboratory analysis, and data curation. AT: Investigation and sample collection. KA: Formal analysis and visualization. AI: Validation, laboratory analysis, and data interpretation. RB: Project administration, and supervision. MB: Data curation, investigation, and writing – review and editing. SN: Statistical analysis, formal analysis, and writing – review and editing. AZ: Supervision, project administration, and final manuscript editing. All authors have read, reviewed, and approved the final manuscript.
